# The effect of sonication-synergistic natural deep eutectic solvents on extraction yield, structural and physicochemical properties of pectins extracted from mango peels

**DOI:** 10.1016/j.ultsonch.2022.106045

**Published:** 2022-05-20

**Authors:** Sijun Chen, Leyan Xiao, Songjie Li, Tingyu Meng, Lu Wang, Weimin Zhang

**Affiliations:** a School of Food Science and Engineering, Hainan University, Haikou 570228, PR China; b Key Laboratory of Food Nutrition and Functional Food of Hainan Province, Hainan University, Haikou 570228, PR China

**Keywords:** DESs, deep eutectic solvents, Bet-CA, betaine-citric acid, ChCl-MaA, choline chloride-malic acid, FFD, full factor design, RSM, response surface methodology, CCD, central composite design, FT-IR, Fourier Transform Infrared spectra, DE, degree of esterification, Mw, molecular weight, Mn, number-average molecular weight, CP, commercial pectin, TG, thermogravimetry, DTG, differential thermogravimetry, Mango peel, Pectin, Deep eutectic solvents, Ultrasound, Physicochemical properties

## Abstract

In this study, eco-friendly deep eutectic solvents (DESs) were used as extracting agents for the first time in the extraction of pectins from mango peel. Two novel green solvents including betaine-citric acid (Bet-CA) and choline chloride-malic acid (ChCl-MaA) were screened, and the extraction conditions were further optimized by full factor design experimental along with RSM. In addition, ultrasound treatment also had an influence on extraction yield, structural and physicochemical properties of extracted pectins. Two DES-extracted pectins had significantly higher yield, larger molecular weight and particles size than HCl-extracted pectin. High intensity ultrasound power enhanced the yield of low-ester pectins, but decreased the molecular weight and particles size of the pectins extracted. Monosaccharide compositions analysis showed that higher content of galacturonic acid (GalA) and larger HG region were observed in two DESs-extracted pectins. Fourier transform infrared spectra (FT-IR) of all pectins extracted were similar, with slight differences. Two DESs-extracted pectins exhibited higher DE values than HCl-extracted pectin. Thermal analysis and zeta potential results showed that HCl-extracted pectin had better stability than ChCl-MaA-extracted pectin. Additionally, HCl-extracted pectin had higher viscosity properties than two DESs-extracted pectins or commercial pectin (CP). Moreover, it was found that HCl-extracted pectin was in a colloid state, while two DESs-extracted pectins or CP were in a flow state. Ultrasound treatment significantly improved the yields of pectin/low-ester pectin. Additionally, ultrasound treatment remarkably decreased the viscosity and viscoelastic properties of the pectins extracted. The results were conducive to our understanding of the relationship between extraction conditions and physicochemical properties of the pectins extracted, which provides theoretical basis for the functional application of mango peel pectins in the food and pharmaceutical industry.

## Highlights

• Eco-friendly deep eutectic solvents (DESs) were used for pectin extraction from mango peels for the first time.
• Two eco-friendly solvents extracted higher pectin yields than conventional hydrochloric acid.
• Process parameters for pectin extraction with two DESs were further optimized by response surface methodology.
• Ultrasound treatment significantly improved the yields of pectin/low-ester pectin.
• Solvents and ultrasound varied the structural and physicochemical properties of pectins.

## Graphical abstract

**Figure:**
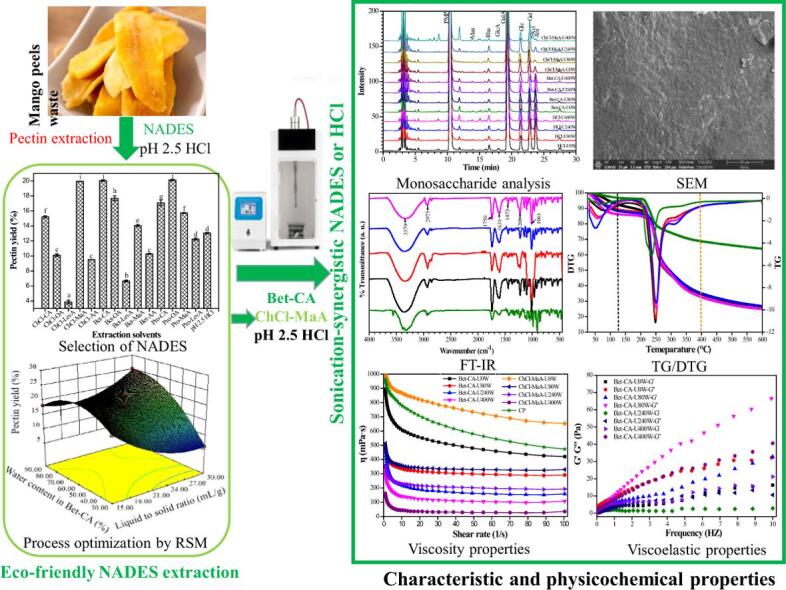

## 1 Introduction

Pectin, as a plant cell wall polysaccharide with complex structure, is usually interacted with the other components, such as cellulose, hemicellulose and protein [1], [2]. Extensive studies have verified that pectins extracted from different materials (citrus peel, apple pomace, berry pomace, passion fruit and sugar beet, etc.) have important application values in foods, cosmetics, and biomedical industries owing to its excellent stability, gelation, emulsification, as well as the function of targeted delivery of bio-active compounds [3], [4]. In addition, pectin polysaccharides have also been proven to have multi-biological activities beneficial for human health, including modulating gut microbiota, ameliorating intestinal inflammation, decreasing the cholesterol level of blood, preventing the development of atherosclerosis and suppressing fat accumulation [5], [6]. It is found that the bio-functional properties of pectin are extremely associated with its structural and physicochemical characteristics, including molecular weight (Mw), galacturonic acid content, degree of esterification (DE), monosaccharide compositions, viscosity, extraction methods, botanical and developmental status [7], [8], [9]. In addition, with the increasing of worldwide demand for pectin, researchers are seeking out high-efficient pectin extraction methods and investigating their physicochemical and functional properties.

Mango (*Mangifera indica* L.), as a type of tropical fruit, is widely produced in many countries, such as China, India, Malaysia and Thailand [3], [10]. In 2019, the annual export volume of mango products in Hainan and Guangxi province of China exported exceeded 2.45 million tons. However, with the huge production of mango, there are considerable amounts of mango peel discarded. Mango peel is rich in pectin, which accounts for 20–30% of dry weight [3], [11]. Extraction of pectin is an important approach for food and biomedical industry to valorize the mango peel wastes. In general, traditional methods for pectin extraction are mainly based on single acid, alkali, and enzymes or their combination with ultrasound/microwave [1], [12]. Acid/alkali extraction methods are labor intensive, time consuming, environment polluting, which may decrease the degree of esterification and the molecular weight of the pectin extracted. Moreover, enzymes or ultrasound/microwave methods normally have low pectin yield and may negatively affect the structural and physicochemical properties of the pectin extracted [13], [14]. Therefore, it is of great importance to explore a novel, high-efficient and eco-friendly method to recovery pectin from mango peel wastes. Currently, natural deep eutectic solvents (NADES), as a new generation of eco-friendly solvents, are considered ideal alternatives to organic solvents in extraction of biopolymers and various plant molecules [15], [16]. Compared with organic solvents, DESs have many merits including simple preparation, non-toxicity, nonvolatility, and good biodegradability, which perfectly matches the conception of “green chemistry” [15], [16]. So far, there is little information about the extraction optimization and physicochemical properties of the pectins extracted from fruit peel wastes by using sonication-synergistic natural DES.

In this study, novel natural deep eutectic solvents were used to extract pectin from mango peels for the first time. Then, the extraction parameters were further optimized by response surface methodology (RSM) by taking pectin yield as an indicator. In addition, the effects of sonication-synergistic solvents on extraction yields, structural and physicochemical properties of the pectins extracted from mango peels were investigated and compared. This study enhances the understanding of the relationship between the extraction methods and physicochemical properties of pectin, and provides theoretical basis for the functional application of pectin in the food and pharmaceutical industry.

## 2 Materials and methods

### 2.1 Materials and chemicals

“Jinhuang” mango species (*Mangifera indica* L.) at maturity was purchased from Hainantainong Biotechnology Co., Ltd. (Haikou, Hainan, China) in September 2020. Firstly, the separated mango peels were soaked in boiling water for 5 min to inactivate enzymes, and then dried in an oven at 60 °C until reaching a constant weight. Subsequently, the dried mango peels were ground into powder by a pulveriser (HC-250Y, Zhejiang, China) and screened using a 40-mesh sieve (diameter 0.425 mm). Twelve monosaccharide standards mannose (Man), ribose (Rib), rhamnose (Rha), glucosamine (GlcN), glucuronic acid (GlcA), galacturonic acid (GalA), glucose (Glc), galactosamine (GalN), galactose (Gal), xylose (Xyl), arabinose (Ara) and fucose (Fuc) were purchased from Sigma-Aldrich Co., Ltd. (Shanghai, China). Commercial pectin (CP) extracted from apple (CAS: 9000-69-5, GalA > 65%) was purchased from Macklin Biochemical Technology Co., Ltd. (Shanghai, China). Chemicals used to prepare DESs were brought from Aladdin Bio-Chem Technology Co., Ltd. (Shanghai, China). Other analytical-grade reagents used in the experiment were purchased from Damou Chemical Co., Ltd. (Tianjin, China).

### 2.2 Preparation of DESs

Fifteen DESs were prepared by using the reported heating method [17]. In short, the mixtures of two components at a suitable molar ratio were put into a round-bottomed flask and stirred at 85 °C to form a transparent and homogeneous liquid (Table 1). In order to improve the extraction efficiency of pectin, 30% (*v/v*) water was added into each DESs to reduce the viscosity. The viscosity of DESs was measured by using a HAAKE MARS 40 type rheometer (ThermoFisher Scientific, Karlsruhe, Germany).

**Table 1:** Lists and physical-chemical properties of DES prepared in this study.

No.	Component A	Component B	Abbreviations	Molar ratio (mol/mol)	Viscosity (mPa·s, 30% water, 25 °C)
1	Choline chloride	Citric acid	ChCl-CA	2:1	246.68 ± 1.38
2	Choline chloride	Oxalic acid	ChCl-OA	1:1	149.16 ± 3.26
3	Choline chloride	Levulinic acid	ChCl-LevA	1:2	270.11 ± 6.87
4	Choline chloride	Malic acid	ChCl-MaA	1:2	140.73 ± 1.95
5	Choline chloride	Acetic acid	ChCl-AA	1:3	222.18 ± 1.77
6	Betaine	Citric acid	Bet-CA	1:2	117.07 ± 3.11
7	Betaine	Oxalic acid	Bet-OA	1:2	119.85 ± 6.69
8	Betaine	Levulinic acid	Bet-LevA	1:1	271.17 ± 5.59
9	Betaine	Malic acid	Bet-MaA	1:2	226.19 ± 2.18
10	Betaine	Acetic acid	Bet-AA	1:3	259.35 ± 4.95
11	l-Proline	Citric acid	Pro-CA	1:2	285.07 ± 2.16
12	l-Proline	Oxalic acid	Pro-OA	1:2	–
13	l-Proline	Levulinic acid	Pro-LevA	1:2	207.65 ± 8.87
14	l-Proline	Malic acid	Pro-MaA	1:2	153.39 ± 8.90
15	l-Proline	Acetic acid	Pro-AA	1:2	–

### 2.3 Extraction and purification of pectin

For the preliminary screening of DESs, dried mango peel powder (0.5 g) was mixed with 15 mL of fifteen types of DESs (containing 30% water) or HCl (pH 2.5) in 20 mL tubes, respectively. Then, these tubes were incubated in a water bath at 90 °C for the pectin extraction. Afterwards, the extracts were centrifuged at 10,000 *×* g for 10 min to collect the supernatant and mix it with two-fold volumes of 95% ethanol. The mixture was stored at refrigerator (4 °C) overnight, followed by centrifugal treatment at 10,000 × g for 15 min, and the resulting precipitated pectin was washed four times with 95% ethanol to fully remove the residual solvents. The recovered pectins were pooled into a dialysis bag with a molecular weight cutoff of 5 kDa for dialysis at 4 °C for 40 h against distilled water, respectively. Lastly, the purified pectins were lyophilized and stored at −20 °C. The yield of pectin was measured by using the following Eq. (1):

(1) $$\text{Pectin}\;\text{yield}\left(\%\right)=\frac{M_{1}}{M_{0}}\times 100$$

where M_1_ denotes the weight of recovered dried pectin; M_0_ represents the weight of dried mango peel powder.

### 2.4 RSM optimization

In order to investigate the effects of the key parameters (pH value of the extraction solvent, extraction time, extraction temperature, L/S ratio and water content in DESs) on pectin yield, a five-factor two-level full factorial design (FFD) experiment was performed.

Based on the above FFD experimental results, the key parameters were further optimized by RSM-based central composite design (CCD) by taking pectin yield as an indicator. A total of 17 experimental runs in a randomized order were examined (Table 2). The response variable pectin yield (Y_Bet-CA_ and Y_ChCl-MaA_) was investigated. The quadratic model for predicting the optimal point was calculated by the following Eq. (2).

(2) $$\text{Y =}\;\eta_{\text{0}}+\sum_{i=1}^{n}\eta_{i}{\text{X}}_{\text{i}}+\sum_{i=1j>2}^{n=1}\sum_{j=2}^{n}\eta_{\mathrm{ij}}{\text{X}}_{\text{i}}{\text{X}}_{\text{j}}+\sum_{i=1}^{n}\eta_{\mathrm{ii}}{\text{X}}_{i}^{2}$$

where X_i_ and X_j_ represent the independent variables; Y_Bet-CA_ and Y_ChCl-MaA_ are the predicted responses; η_0_, η_i_, η_ii_ and η_ij_ represent the intercept, linear, quadric and interaction coefficients, respectively; n is number of variables.

**Table 2:** Experimental results obtained for the experimented and predicted responses with the pectin from mango peel.

Exp.	Independent variables			Y_1_ (%)		Independent variables			Y_2_ (%)	
	A: pH	B: L/S (mL/g)	C: WC (%)	Exp.	Pred.	D: pH	E: T (°C)	F: t (min)	Exp.	Pred.
1	1.85 (0)	30.00 (+1)	30.00 (−1)	6.07 ± 0.05	6.26	1.40 (+1)	100.00 (+1)	90.00 (0)	16.95 ± 0.01	17.67
2	1.85 (0)	22.50 (0)	60.00 (0)	22.59 ± 0.09	18.32	0.90 (0)	70.00 (−1)	60.00 (−1)	11.71 ± 0.004	13.35
3	2.70 (+1)	22.50 (0)	90.00 (+1)	10.52 ± 0.02	10.78	1.40 (+1)	70.00 (−1)	90.00 (0)	12.25 ± 0.007	10.22
4	1.00 (−1)	22.50 (0)	90.00 (+1)	26.22 ± 0.01	26.37	0.90 (0)	100.00 (+1)	60.00 (−1)	22.36 ± 0.004	21.24
5	2.70 (+1)	22.50 (0)	30.00 (−1)	10.51 ± 0.03	10.36	0.40 (−1)	85.00 (0)	60.00 (−1)	19.14 ± 0.08	18.23
6	1.85 (0)	22.50 (0)	60.00 (0)	15.72 ± 0.002	18.32	0.90 (0)	70.00 (−1)	120.00 (+1)	14.43 ± 0.01	15.54
7	1.85 (0)	15.00 (−1)	90.00 (+1)	18.21 ± 0.004	18.02	0.90 (0)	85.00 (0)	90.00 (0)	19.03 ± 0.01	17.06
8	1.85 (0)	15.00 (−1)	30.00 (−1)	18.23 ± 0.03	18.43	0.90 (0)	85.00 (0)	90.00 (0)	15.42 ± 0.01	17.06
9	1.85 (0)	22.50 (−1)	60.00 (0)	17.74 ± 0.01	18.32	0.90 (0)	85.00 (0)	90.00 (0)	15.05 ± 0.01	17.06
10	1.85 (0)	22.50 (0)	60.00 (0)	15.87 ± 0.01	18.32	1.40 (+1)	85.00 (0)	120.00 (+1)	10.70 ± 0.02	11.61
11	2.70 (+1)	15.00 (−1)	60.00 (0)	19.58 ± 0.007	19.50	0.90 (0)	85.00 (0)	90.00 (0)	16.96 ± 0.004	17.06
12	1.00 (−1)	22.50 (0)	30.00 (−1)	8.80 ± 0.01	8.54	0.90 (0)	85.00 (0)	90.00 (0)	18.84 ± 0.02	17.06
13	1.00 (−1)	30.00 (+1)	60.00 (0)	23.69 ± 0.03	23.76	0.40 (−1)	85.00 (0)	120.00 (+1)	27.48 ± 0.03	27.09
14	1.00 (−1)	15.00 (0)	60.00 (0)	28.93 ± 0.01	28.96	0.40 (−1)	100.00 (+1)	90.00 (0)	25.75 ± 0.02	27.77
15	2.70 (+1)	30.00 (+1)	60.00 (0)	19.48 ± 0.01	19.45	0.40 (−1)	70.00 (−1)	90.00 (0)	19.32 ± 0.05	18.59
16	1.85 (0)	30.00 (+1)	90.00 (+1)	25.15 ± 0.004	24.93	1.40 (+1)	85.00 (0)	60.00 (−1)	14.86 ± 0.01	15.24
17	1.85 (0)	22.50 (0)	60.00 (0)	19.68 ± 0.02	18.32	0.90 (0)	100.00 (+1)	120.00 (+1)	25.91 ± 0.03	24.27

### 2.5 Characterization of the pectins extracted under ultrasound power

In addition to traditional process parameters, ultrasound power also greatly affects the yield, structural and physicochemical properties of the pectins extracted. First, dried mango peels powder was mixed with Bet-CA and ChCl-MaA, respectively. Ultrasound was applied using a probe (25.4 mm diameter) system (Qsonic Q700, USA) with an maximum ultrasound power of 700 W and frequency of 20 kHz, and equipped with a temperature, amplitude, time, and pulse control system. Under the above optimum conditions, the pectin extraction was performed by using the selected solvents under different ultrasound powers (0, 80, 240 and 400 W). The distance between the ultrasound probe tip and the bottom of the cup was controlled by 2 cm. The pectins were recovered based on the method described in Section 2.3.

#### 2.5.1 Pectin yield

Pectin yield was calculated according to the method described in Section 2.3.

#### 2.5.2 Degree of esterification

The titrimetricrically method reported by Kazemi, Khodaiyan, and Hosseini, (2019) [18] was used to determine the degree of esterification (DE) of the recovered pectin. The DE value was calculated using the following Eq. (3):

(3) $$\text{DE}\left(\%\right)=\frac{V_{2}}{V_{1}+V_{2}}\times 100$$

#### 2.5.3 SEC-MALLS analysis

The pectin samples were dissolved in 0.1 M NaCl solution to prepare a pectin concentration of 1 mg/mL. Before HPLC analysis, the supernatant was centrifuged at 10,000 *×* g for 30 s at 4 °C and subsequently filtered via a cellulose acetate membrane (0.45 μm of pore size, Millipore). Molecular parameters (Mw, Mn, and Mw/Mn) of pectin samples were determined by a HPLC system (Waters e2695, MA, USA) equipped with a multi-angle laser-light scattering (MALLS) and a differential refractive index detector (Waters, MA, USA). The flow rate was set to 0.8 mL/min. The mobile phase was 0.1 M NaCl solution. The data was processed by using ASTRA5.3.4.20 software [19].

#### 2.5.4 Particle size, PDI, zeta-potential and conductivity

Pectins samples (100 mg) were resolved in 10 mL of deionized water. The particle size, PDI, zeta-potential and conductivity of pectin solutions were measured by Malvern Panalytical Zetasizer Nano ZS instrument (Malvern instrument Ltd., MA, USA).

#### 2.5.5 Monosaccharide analysis

Pectin powder (15 mg) was mixed with 5 mL of 2 M trifluoroacetic acid, and then hydrolysed in a sealed glass tube at 100 °C for 3 h under nitrogen. After cooling down to room temperature, the hydrolysate was adjusted to pH 7 with 0.3 M NaOH and diluted to 4 mL. 100 μL of the diluted hydrolysate or fifteen monosaccharide solution was mixed with 400 μL of 0.5 M 1-phenyl-3-methyl-5-pyrazolone-methanol (PMP-methanol) solution, and then heated in a hot water bath at 70 °C for 30 min [19]. By adding 0.3 M HCl, the reaction solution was adjusted to pH = 7. Afterwards, the solution was extracted three times with 1 mL chloroform to remove PMP reagent, and the derivatized solution was filtered via a 0.45 μm cellulose acetate membrane before performing the monosaccharide analysis with a Agilent 1100 HPLC system coupled with a Diode array detector. The Agilent ZORBAX Eclipse Plus C18 column (250 mm × 4.6 mm, 5 μm) (Agilent, MA, USA) was used for separating the monosaccharide. The mobile phase consisted of 0.1 M sodium phosphate buffer (phase A, pH 6.7) and acetonitrile (phase B). The gradient elution of mobile phase was conducted as follows: 0 min, 86% A; 0–9 min, 83% A; 9–28 min, 78% A; 28–29 min, 50% A, 29–31 min, 50% A; 31–32 min, 86% A; 32–40 min, 86% A. The flow rate, injection volume, column temperature and UV detection wavelength were set to 1 mL/min, 5 μL, 30 °C and 250 nm, respectively.

### 2.6 FT-IR spectrum and thermal analysis

The extracted pectin powder was ground with potassium bromide (KBr, 1:100) and compressed together. Then, FTIR spectrum characteristics of the pectin samples were analyzed by Fourier transform infrared spectrum spectrometer (Thermo Scientific, MA, USA) at a wavelength range of 4000–400 cm^−1^ with a high resolution of 4 cm^−1^.

The thermodynamic characteristic (TG/DTG analysis) of the pectin samples extracted was analyzed by a TG 209 F3 thermal analyzer (NETZSCH Scientific Instruments, Germany). The pectin samples (10 mg) were sealed in aluminium crucibles and heated from 60 °C to 600 °C at a linear rate of 10 °C min^−1^ under high-purity nitrogen flow.

### 2.7 Scanning electron microscopy

The pectin samples were fixed on aluminum stabs with thin gold layer. Then, their morphological characteristics were observed and photographed by using a Verios G4 UC type scanning electron microscopy (SEM; ThermoFisher scientific Ltd., MA, USA) with an accelerating voltage of 5 kV and amplification of 2000×.

### 2.8 Rheological properties

#### 2.8.1 Apparent viscosity

According to the method described by Huang et al. [20], the pectin powder was dispersed in deionized water to reach the concentration of 3% (*w/v*) for the rheological analysis (pH = 3.80). The flow behaviors of the pectin solutions were analyzed by using an HAAKE MARS40 Rheometer (Thermo Fisher, MA, USA) and represented by viscosity curves over a shear rate from 1 to 100 s ^−1^ at 25 °C. The data measured data were fitted to the Herschel-Bulkley model based on the Eq. (4) [21].

(4) $$\tau =\varepsilon_{0}+K{\left(\gamma \right)}^{n}$$

where τ and ε_0_ represent the shear stress and the yield stress, respectively. γ is shear rate. K and n represent adimensinal constants (consistency index and flow behavior index).

#### 2.8.2 Viscoelastic properties

The viscoelastic properties of the pectin solutions at concentration of 3% (*w/v*) were determined by using an HAAKE MARS40 Rheometer (Thermo Fisher, MA, USA). Before the dynamic oscillation measurements, the linear viscoelastic region was evaluated from 0.001% to 100% at a stable frequency of 1 Hz [22]. Afterwards, the frequency sweep over the range from 0.1 to 10 Hz at a constant stress of 5 Pa and under temperature of 25 °C was carried out to monitor the change in the storage (G′) and loss modulus (G″) of the prepared pectin solutions.

### 2.9 Statistical analysis

FFD, CCD-based RSM and regression coefficient analysis were conducted by using Design Expert software version 10.0 (Stat-Ease Inc., USA). The statistics analysis were performed by two-way ANOVA using IBM SPSS Statistics software (IBM Corp., NY, USA). All tests were performed in three replicates and results were represented as means ± standard deviations. The difference was considered significant when *p* < 0.05.

## 3 Results and discussion

### 3.1 Screening of high-efficient pectin extraction solvents

**Fig. 1:**
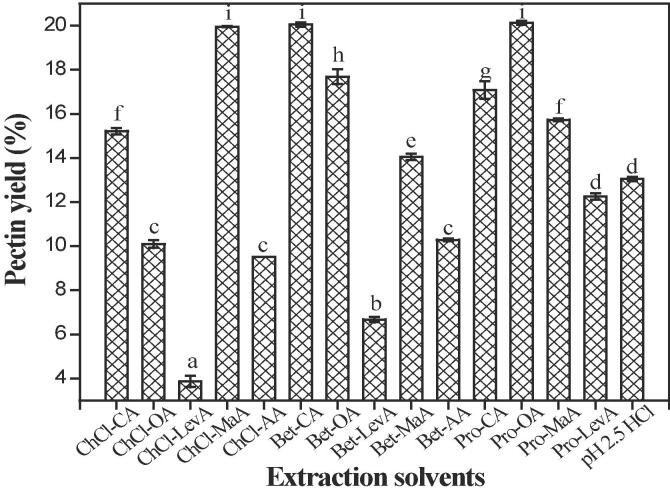
Yields of pectins extracted from mango peels using different DESs. The values with different lowercase letters (a–h) are significantly different (*p* < 0.05). The extraction conditions are as follow: water content in DESs of 30%, extraction temperature of 90 °C, liquid-to-solid ratio of 30 mL/g, extraction time of 120 min. The HCl (pH 2.5) extraction is regarded as the control.

Generally, the viscosity, solubility, polarity and surface tension were key factors influencing the extraction efficiency of polar/non-polar active molecules when using DESs as extracting agents [23], [24]. In this work, three different types of DESs were chosen as the extractants to extract pectin from mango peels for the first time. Conventional HCl (pH = 2.5) was adopted as the control [25]. As shown in Fig. 1, it can be observed that most of DESs showed higher pectin extraction yield than conventional HCl. Among these tested DESs, Bet-CA (20.05%), Pro-OA (20.13%) and ChCl-MaA (19.95%) exhibited better pectin extraction ability than conventional HCl (13.05%). Although Pro-OA exhibited good extraction efficiency for pectin, it is difficult to be applied in large-scale industrial production as it is easily crystallized at room temperature. In addition, we found that fifteen DESs had remarkably different viscosity. From Table 1, DESs (Bet-CA-117.07 mPas, ChCl-MaA-140.73 mPas and Bet-OA-119.85 mPas) with low viscosity showed higher pectin yield than the other DESs with high viscosity, which was consistent with the results reported by Cao et al. [23] who determined that ChCl-EG with low viscosity had higher extraction yield of polysaccharides than the other DESs with high viscosity. This may be because that high viscosity of extracting agents not only impede the diffusion of target compounds in the solution, but also greatly affect energy- and mass- transfer efficiency, which is not beneficial to the extraction of pectin polysaccharides [23], [26]. Therefore, two novel extracting agents (Bet-CA and ChCl-MaA) were selected for the subsequent process optimization.

### 3.2 Process optimization and validation of predictive models

A five-factor two-level FFD experiment was conducted to evaluate the influence of process conditions on the extraction yield of pectin from mango peels using two DES solvents (Bet-CA and ChCl-MaA) (Table S1). The key factors significantly affecting pectin extraction yield can be explained by the pareto chart of the standardized effects (Fig. S1). Results showed the extraction parameters had remarkable influences on pectin yield. For Bet-CA, pH value of the extraction solvents (A), L/S ratio (B) and water content in Bet-CA (C) were determined as the key independent variables on pectin yield (Fig. S1A). For ChCl-MaA, pH value of the extraction solvents (D), extraction temperature (E), and extraction time (F) were the key independent variables (Fig. S1B). These key factors were considered in further RSM-based optimization of the extraction parameters of pectin.

Table 2 shows the independent variables, measured values and predicted values. The measured values for pectin yield (6.26–28.96% for Bet-CA and 10.70–27.48% for ChCl-MaA) indicate that the optimization of extraction parameters is of great importance for obtaining high content of pectin. The following second-order polynomial equations (5), (6) were obtained by multiple regression analyses:

(5) $${\text{Y}}_{\text{Bet}-\text{CA}}=26.63=3.42{\text{A - 1.34B + 4.56C + 1.24AB - 4.35AC + 4.77BC + 0.88A}}^{\text{2}}+3.78B^{2}-5.18C^{2}$$

(6) $${\text{Y}}_{\text{ChCl - MaA}}=27.06-4.62D+4.16E+1.31F-0.43DE-3.12DF++0.21EF+0.47D^{2}+1.03E^{2}+0.5IF^{2}$$

**Fig. 2:**
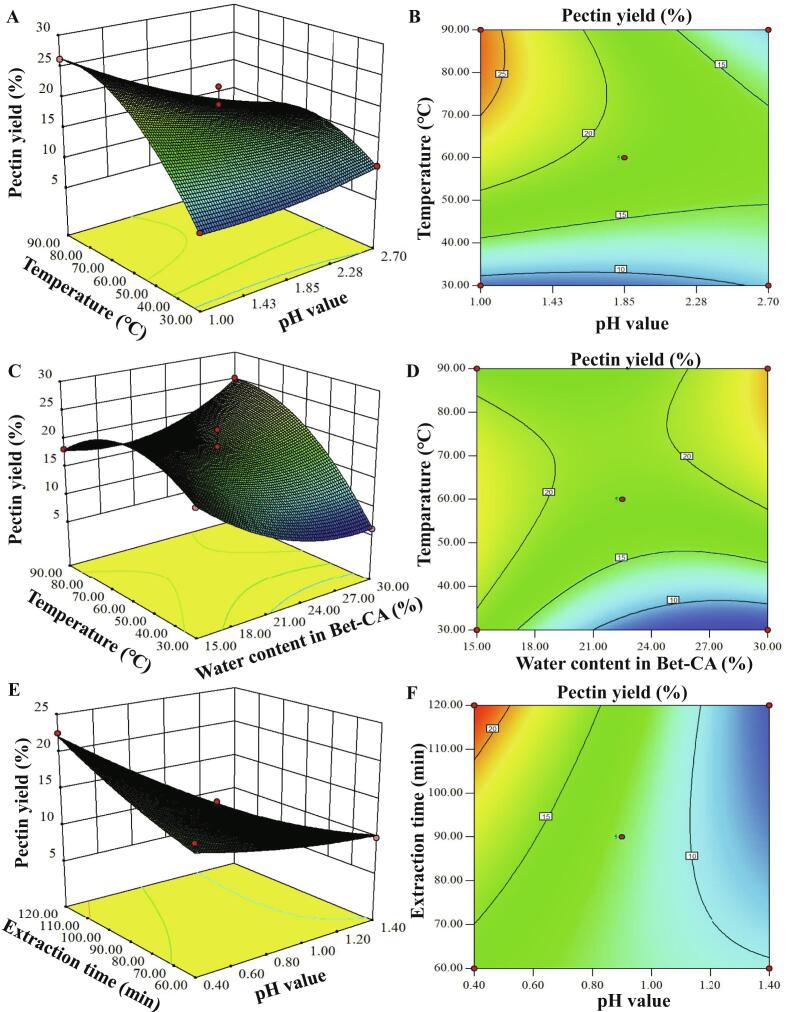
3D response surface plots indicating the interactions between process conditions for extraction of pectin from mango peels using betaine-citric acid (Bet-CA) and choline chloride-malic acid (ChCl-MaA) as extracting agents.

Table 3 shows the results of ANOVA analysis. It can be seen that the proposed model was in accordance with the Tukey’s HSD (*p* < 0.01) with no significant lack of fit (*p* > 0.05). It was confirmed that the models well matched with the experimental value for two DESs. High values of R^2^ (0.9480 for Bet-CA and 0.9184 for ChCl-MaA) were observed, indicating high degree of fit between predicted and measured values. In addition, the adequacy precision over 4.0 reflected a desirable and credible result of signal-to-noise ratio [16], [27]. In this work, high adequacy precision (13.497 for Bet-CA and 10.568 for ChCl-MaA) implied that the measured value was precise and credible for the optimization of process parameters. With regard to Bet-CA, the pH value of Bet-CA (A) and the water content in Bet-CA (C) had significant (*p* < 0.01) effects on pectin yield, whereas the liquid-to-solid ratio had no significant effect on the pectin yield. The quadratic coefficients (B^2^ and C^2^) had significant effects (*p* < 0.01) on pectin yield. In addition, the interaction between pH value of Bet-CA and water contents in Bet-CA (AC) and that between liquid-to-solid ratio and water content in Bet-CA (BC) had significantly influence on pectin yield (*p* < 0.01). With regard to ChCl-MaA, the pH value of ChCl-MaA (D) and the extraction temperature (E) had extremely significant effects (*p* < 0.001) on pectin yield, but the extraction time had no influence on pectin yield. All quadratic coefficients had no significant effects on the pectin yield (*p* > 0.05). Additionally, the pH value of ChCl-MaA and the extraction time (DF) had a significant interactive effect on pectin yield (*p* < 0.05). According to Eqs. (4), (5), the 3D response surface plots were constructed. From Fig. 2AB, it can be found that the water contents in Bet-CA (C), the interaction between the pH value of Bet-CA and liquid-to-solid ratio (AB) and that between liquid-to-solid ratio and water content in Bet-CA (BC) posed positive effects on pectin yield. However, the pH value of Bet-CA (A), the liquid-to-solid ratio (B) and the interaction between the pH value of Bet-CA and the water content in Bet-CA (AC) displayed negative effects on pectin yield. Yan et al. (2021) reported that the pH value of extraction solvent was an important factor influencing the extraction yield, structural, and rheological properties of citrus pectin [28]. With regard to ChCl-MaA, it was observed that the extraction temperature (E), the extraction time (F) and the interaction between extraction temperature and extraction time (EF) had positive effects on pectin yield. However, the pH value of ChCl-MaA (D), the interaction between the pH value of ChCl-MaA and the extraction temperature (DE) and that between the pH value of ChCl-MaA and the extraction time (DF) showed negative effects on pectin yield (Fig. 2C). It was reported that the increase in temperature or extraction time at acidic environment may disrupt the cross-link structures of cell wall polymers, and enhance the release of pectin polysaccharides [29], [30]. In addition, the increase in temperature can enhance the mass transfer and the diffusion rate, but decline the viscosity and surface tension of ChCl-MaA, thereby increasing the extraction yield of pectin [31].

**Table 3:** Analysis of the variance of the fitted second-order polynomial models and validation of the responses under the optimal conditions.

Source	DF	Some of squares	Mean square	F-value	*p*-value
Y_BC_ optimization					
Model	9	614.88	68.32	14.17	0.0010^***^
A	1	93.51	93.51	19.40	0.0031^**^
B	1	14.38	14.38	2.98	0.1278
C	1	166.53	166.53	34.54	0.0006^***^
AB	1	6.12	6.12	1.27	0.2971
AC	1	75.78	75.78	15.72	0.0054^**^
BC	1	91.03	91.03	18.88	0.0034^**^
A^2^	1	3.26	3.26	0.68	0.4380
B^2^	1	60.05	60.05	12.45	0.0096^**^
C^2^	1	112.97	112.97	23.43	0.0019^**^
Lack-of-Fit	3	0.41	0.14	0.016	0.9967 ^ns^
Pure Error	4	33.34	8.34		
Total	16	648.63			
R^2^	0.9480				
Adj R^2^	0.9113				
Pred R^2^	0.9097				
Adeq precision	13.497				
Y_CM_ optimization					
Model	9	369.57	41.06	8.76	0.0046^**^
D	1	170.63	170.63	36.40	0.0005^***^
E	1	138.19	138.19	29.48	0.0010^***^
F	1	13.64	13.64	2.91	0.1318
DE	1	0.75	0.75	0.16	0.7013
DF	1	39.06	39.06	8.33	0.0234*
EF	1	0.18	0.18	0.038	0.8517
D^2^	1	0.94	0.94	0.20	0.6677
E^2^	1	4.48	4.48	0.95	0.3611
F^2^	1	1.09	1.09	0.23	0.6446
Lack-of-Fit	3	19.04	6.35	1.84	0.2796 ^ns^
Pure Error	4	13.77	3.44		
Total	16	402.38			
R^2^	0.9184				
Adj R^2^	0.8136				
Pred R^2^	0.8037				
Adeq precision	10.568				

ns, Not significant (*p* > 0.05).

* , ^**^, and ^***^, Significant at *p* ≤ 0.05, *p* ≤ 0.01 and *p* ≤ 0.001, respectively.

In order to validate the model, the additional confirmation tests were performed under the optimal conditions. For Bet-CA extraction, the predicted value (27.08%) was in agreement with measured value (27.62%) under extraction conditions of pH = 0.85, liquid-solid ratio of 27: 1, extraction temperature of 80 °C, water content of Bet-CA at 70%, and extraction time of 90 min. For ChCl-MaA extraction, under the optimum conditions (under extraction conditions of pH = 0.40, extraction temperature of 85 °C, extraction time of 120 min, water content of ChCl-MaA at 70%, liquid-solid ratio of 27:1), the measured pectin yield (30.01%) was consistent with the predicted value (29.31%). The results indicated the high accuracy and feasibility of the model.

### 3.3 Characterization of pectins extracted with different methods

#### 3.3.1 Pectin yield

As shown in Table 4, the extraction solvent used significantly affected pectin extraction yield. Under the above optimal conditions without ultrasound treatment, two DESs exhibited relatively higher pectin extraction yields (27.62% for Bet-CA and 30.01% for ChCl-MaA) than conventional HCl (13.17%). Our results can be compared to the yields of pectin extracted from mango peels with those reported literatures, which contain approximately 6.2–8.1% and 10.33% extraction yield (Table 5). The pectin yields extracted by using conventional HCl was in agreement with a previous study that indicated a 13.04% maximum yield [3]. In this work, it can be found that two selected DESs showed a higher extraction yields of pectins extracted from mango peels than those reported in other literatures [32], [33], [34], [35], [36] (Table 5). Currently, DESs are widely applied in high-efficient extraction of some small molecules including phenolic, flavonoids, and alkaloids etc. Only few studies have reported that DESs can be also used to extract macromolecular compounds, such as protein, polysaccharides, and DNA, etc. [19], [37], [38]. In this study, the DESs used exhibited excellent extraction efficiency for pectin from mango peels. Suitable DESs can enhance the extraction of the pectins attached to the hemicellulose/cellulose by solubilising the plant cell wall structures [38], [39]. Gao et al. (2020) reported that DES ChCl-EG (Choline chloride-ethylene glycol) increased the extraction of polysaccharides from *Camellia oleifera* Abel. seed cake [23]. However, Qin Liew et al. [40] found that citric acid showed a better extraction performance than lactic acid-based DESs in pectin extraction, which was not consistent with the results of our study. It may be due to that the type, pH value and viscosity of DESs significant affected the extraction efficiency of pectin [23], [40]. In addition, ultrasound treatment is also an important factor affecting the extraction yield of pectin. It can be found that high intensity ultrasound treatment evidently enhanced the extraction yield of pectins no matter what kinds of solvents were used, which is consistent with the results reported from Sengar et al. [41] who determined ultrasound wave enhanced the extraction yield of pectin from tomato processing waste. As we all know, ultrasound wave can not only destroy the cross-linking bonds between pectin and cell wall structure, but also accelerate the energy- or mass- transfer, thereby leading to the release of more bio-active compounds. Many researchers have definitely verified that ultrasound procedure can be applied in the extraction of pectin from various plant-based resources with less energy consumption and higher efficiency [14], [15]. In short, the extraction yield of pectin from mango peels is remarkably influenced by extracting agents and ultrasound power.

**Table 4:** The yields, Mw, Mn, DE value, particle size, zeta potential and conductivity of pectin recovered from mango peels by different methods.

Abbreviations	Pectin yield (%)	DE (%)	Mw (KDa)	Mn (KDa)	Mw/Mn	Particle size (nm)	Zeta Potential (mV)	PDI	Conductivity (mS/cm)
HCl-U0W	13.17 ± 1.21Aa	65.55 ± 1.16Ac	706.14 ± 1.03Be	557.41 ± 8.78Be	1.360	1004.33 ± 83.84Ad	–23.27 ± 1.59Ac	0.43 ± 0.07Aa	0.0330 ± 0.0001Aa
HCl-U80W	15.21 ± 0.92Ab	59.03 ± 2.11Ab	676.16 ± 5.12Be	497.13 ± 7.17Cd	1.267	721.57 ± 50.37Ac	–23.67 ± 0.91Ac	0.51 ± 0.05Aa	0.0378 ± 0.0041Aa
HCl-U240W	16.75 ± 0.37Ac	58.44 ± 1.35Ab	612.10 ± 3.17Bb	407.63 ± 5.31Bc	1.502	657.50 ± 11.98Ab	−21.00 ± 0.56Ac	0.49 ± 0.04Aa	0.0336 ± 0.0001Aa
HCl-U400W	17.37 ± 1.08Ac	55.10 ± 0.51Aa	619.97 ± 7.38Bb	421.97 ± 8.13Ac	1.469	619.00 ± 25.94Aa	–22.23 ± 1.96Ac	0.44 ± 0.02Aa	0.0524 ± 0.0010Bb
Bet-CA-U0W	27.62 ± 3.52Bd	83.43 ± 4.76Bf	781.67 ± 6.32Cf	715.55 ± 6.71Cf	1.392	1676.67 ± 23.66 Ci	−17.07 ± 0.42Bb	0.65 ± 0.06Bb	0.0653 ± 0.0006Bc
Bet-CA-U80W	30.50 ± 6.05Be	79.47 ± 1.31Be	686.33 ± 5.41Ce	417.52 ± 2.37Bc	1.644	1577.66 ± 26.16Ch	−16.97 ± 0.80Bb	0.61 ± 0.08Cb	0.0790 ± 0.0010Bc
Bet-CA-U240W	33.31 ± 3.38Bf	76.56 ± 1.28Bd	631.50 ± 3.72Cb	436.41 ± 6.15Cc	1.447	1472.33 ± 8.49Cg	−15.60 ± 0.30Ba	0.50 ± 0.02Ba	0.0857 ± 0.0012Bd
Bet-CA-U400W	36.76 ± 6.23Bg	75.05 ± 1.15Bd	626.64 ± 9.17Bb	458.89 ± 9.64Bc	1.366	1357.00 ± 53.36Cf	−15.30 ± 0.62Ca	0.58 ± 0.09Ba	0.0870 ± 0.0010Bd
ChCl-MaA-U0W	30.01 ± 1.38Ce	87.06 ± 1.97Cg	641.47 ± 3.65Ac	386.68 ± 2.52Ab	1.659	1503.33 ± 38.89Bg	−16.70 ± 0.53Bb	0.65 ± 0.09Bb	0.0800 ± 0.0010Ce
ChCl-MaA-U80W	35.40 ± 2.72Cg	84.55 ± 1.44Cf	651.53 ± 4.41Ad	392.36 ± 3.72Ab	1.661	1535.67 ± 63.64Bh	−17.13 ± 0.72Bb	0.52 ± 0.06Ba	0.0871 ± 0.0015Ce
ChCl-MaA-U240W	36.36 ± 3.48Cg	79.46 ± 1.08Be	560.45 ± 3.78Aa	356.71 ± 8.76Ab	1.571	1436.33 ± 44.77Bg	−16.67 ± 1.19Cb	0.66 ± 0.01Bb	0.0817 ± 0.0006Bd
ChCl-MaA-U400W	38.72 ± 5.61Ch	71.17 ± 1.99Bd	567.03 ± 6.89Aa	455.63 ± 6.12Bc	1.244	1227.33 ± 53.03Be	−17.20 ± 0.26Bb	0.72 ± 0.02Cc	0.0840 ± 0.0010Bd
CP	–	64.95 ± 1.47c	549.38 ± 2.14a	286.03 ± 3.13a	1.920	1036.80 ± 50.31d	−38.33 ± 1.12d	0.39 ± 0.03a	0.0330 ± 0.0020a

**Table 5:** Comparison of yields and DE value of pectins extracted by various methods from mango peels.

Samples	Extraction solvents	Extraction methods	Pectin yield	DE	References
(*M. indica* var.Ataulfo) Mango peel	Acidic water	Microwave at 800 W; 130 °C/30 min	25.01%	**–**	[33]
Ubá mango peel	Acidic water	Temperature at 85.4–97 °C; Extraction time at 35–48 min; pH at 1.66–2.4	18.8–32.10% for per gram alcohol insoluble residue	62.20–86.2% for per gram alcohol insoluble residue	[34]
Mango peel	0.1 N HCl	Temperature at 90 °C; Extraction time at 1.73 h; pH at 1.36	23.69%	86.96%	[35]
(‘Tainong No. 1’) mango peel	Citric acid–water	Ultrasound power at 500 W at 20 kHz; pH at 2.5; 80 °C/15 min; liquid–solid ratio at 40: 1	16.70–17.15%	85.43–88.38%	[36]
Mango (*M. indica* cv. Nam Dokmai) peels	Acidic water	Microwave at 500 W for 20 min at 2450 MHz; pH at 1.5	10.33%	76.47%	[3]
“Jinhuang” mango peel	HCl-water	Temperature: 90 °C; Extraction time: 1.73 h; pH: 1.36	17.37%	55.10%	In this study
“Jinhuang” mango peel	DES (betaine-citric acid)	pH at 0.85, liquid–solid ratio at 27: 1, extraction temperature at 80 °C, Water content of Bet-CA at 70%; extraction time at 90 min and ultrasound power at 320 W	36.76%	75.05%	In this study
“Jinhuang” mango peel	DES (choline chloride-malic acid)	pH at 0.40; extraction temperature at 85 °C; extraction time: 120 min; water content of ChCl-MaA at 70%; liquid–solid ratio at 27:1; ultrasound power at 400 W	38.72%	71.17%	In this study

#### 3.3.2 Degree of esterification

Degree of esterification (DE) determines the physicochemical properties of the pectins extracted. Normally, pectins can be divided into two types, including high methoxyl pectin (HMP, DE > 50%) and low methoxyl pectin (LMP, DE < 50%). Table 4 shows that the DE value of the pectins extracted from mango peels ranges from 55.10% to 87.06%. From the perspective of DE value, all pectins recovered from mango peels can be classed as HMP. It was found that the DE value (55.10–65.55%) of HCl-extracted pectin was significant lower than that of Bet-CA-extracted pectin (75.05%–83.43%) and ChCl-MaA-extracted pectin (71.17%–87.06%). This might be due to that strong acid caused the demethylation and fragmentation of pectin polygalacturonic chains, leading to the formation of more pectin with low DE values [19], [42]. The DE values of the pectins extracted using two DESs in the present study were similar to the DE value of the pectin extracted using HCl (pH = 1.5, 79%) in the previous study of Chaiwarit et al. [3], while the pectins extracted by HCl with pH = 2.5 (55.10–65.55%) were found to have a lower DE value than that extracted by HCl with pH = 1.5. In addition, the DE value of the extracted pectin remarkably declined as the ultrasound power increased (Table 4). Yan et al. (2021) have also reported that enhanced β-elimination and de-methylation reactions caused by ultrasound treatment can lead to dramatic breakages of pectin side chains and methoxyl groups [28]. Similar findings were observed by Ogutu and Mu (2017) and Larsen et al. (2021) who reported that the β-elimination reaction induced by ultrasound waves caused the cleavage of pectin side chain, resulting in the decrease of the DE value of the extracted pectin [43], [44].

#### 3.3.3 SEC-MALLS analysis

In general, molecular weight-average (Mw), number-average molecular weight (Mn) and polydispersity had remarkably influences on the bio-activities and potential applications of the pectins extracted [19], [45]. As demonstrated in Table 4, the pectins extracted had different values of Mw (560.450–781.665 kDa) and Mn (356.709–715.553 kDa), which were much greater than that of commercial pectin (Mw-549.38 kDa and Mn-286.01 kDa). Without ultrasound treatment, Bet-CA-extracted pectin showed the largest values of Mw (781.665 kDa) and Mn (715.553 kDa), followed by ChCl-MaA-extracted pectin with Mw (641.474 kDa) and Mn (386.679 kDa), and HCl-extracted pectin indicated the smallest values of Mw (549.382 kDa) and Mn (286.034 kDa). Similar results were reported by Chen and Lahaye (2021) who determined that the pectins extracted from apple pomace by DESs CC:LA had remarkably higher Mw than that extracted by acid water [37]. Additionally, the Mw and Mn values of pectins extracted decreased with the increase of ultrasound intensity no matter what kinds of solvents were used. The Mw and Mn of pectins extracted under different ultrasound powers were in line with the results of the previous studies in which ultrasound cavitation was found to be capable of breaking the glycosidic bond of the pectin polysaccharides, thereby producing more pectins with low Mw [28]. It is well known that a larger Mw/Mn of pectin solution indicates a broader distribution of molecular weight [31]. Moreover, we found that the pectins extracted from mango peel had different values of Mw/Mn (ranging from 1.244 to 1.661), which was remarkably lower than that of CP (1.920) and other byproducts, such as water-soluble peach gums [46], citrus peel wastes [47] and potato pulp [29]. The results verify that the pectins extracted by DESs or HCl have relatively more unified distribution of molecular weight than CP.

#### 3.3.4 Average particle size

Normally, particle size indirectly depicts the average size of pectin polymers, which is an indispensable factor affecting the functional properties of pectin polysaccharides. Without ultrasound treatment, it was found that the average particle size of the pectin extracted by Bet-CA (1676.67 nm) and ChCl-MaA (1503.33 nm) was evidently bigger than that extracted by conventional HCl (1004.33 nm). Studies have verified that strong acidic treatment may lead to the cleavage of pectin polymer chain, thereby producing more pectin with smaller particle size [40]. In addition, the average particle size of pectin recovered remarkably declined with the increase of ultrasound power no matter what kinds of solvents were used, which was consistent with the results of the previous study who has reported that ultrasound cavitation or microwave heating treatment can degrade pectin polymer chain, and thereby led to release of more pectin with smaller particle size [48].

#### 3.3.5 PDI, zeta-potential and conductivity

PDI, zeta-potential and conductivity are also associated with the stability of pectin colloidal distribution. Generally, smaller molecular weight distribution would lead to a higher absolute value of zeta potential and a more stable colloidal system. As shown in Table 4, it can be observed that the zeta potential values of all pectin solutions were negative, indicating that the mango peel pectin was an acidic pectin polysaccharide [19]. In addition, the absolute values of zeta potential of two DESs-extracted pectins ranged from 15.30 to 17.20 mV, which were significant lower than that of HCl-extracted pectin (from 21.00 to 23.67 mV). It may be because that HCl-extracted pectin had smaller molecular weight (612.095–706.141 KDa) and particle size distribution (619.00–1004.30 nm) than DES-extracted pectin. The results also confirmed that HCl-extracted pectin solution was more stable than DES-extracted pectin. A lower PDI value normally means a better dispersion of the solution system and a lower tendency to agglomerate [49]. In this study, the PDI value of DESs-extracted pectin solution was slightly higher than that of HCl-extracted pectin, which indicates HCl-extracted pectin had better particle dispersion than DESs-extracted pectin. Many studies also confirmed that the conductivity of the solution may change with the stability of solution over time. In general, a smaller molecular distribution can generate a low conductivity and a more stable system [50]. It was found that the conductivity of HCl-extracted pectin ranged from 0.0330 to 0.0524 mS/cm, which were remarkably lower than that of DES-extracted pectin (from 0.0653 to 0.0871 mS/cm), indicating that HCl-extracted pectin had better stability than two DESs-extracted pectins. However, ultrasound treatment has no significant effects on PDI, zeta-potential and conductivity of the same solvent-extracted pectins.

#### 3.3.6 Monosaccharide composition

Monosaccharide composition of pectin polysaccharides has a great influence on the structures and functional properties of the pectins extracted. The monosaccharide composition and content of pectins extracted by different methods are shown in Fig. S2 and Table 6, respectively. The same monosaccharide composition of Man, Rha, GlcA, GalA, Glc, Gal, Xyl, and Ara, was observed for all the extracted pectins. In particular, GalA, Glc, and Gal were three main monosaccharide components for all pectins samples. The proportion of GalA in all extracted pectins ranged from 61.54% to 77.47%, which was similar to that of the quality standard of commercially pectin (GalA ≥ 65%, *w/w*) recommended by Joint Food and Agriculture Organization of the United Nations (FAO)/the World Health Organization (WHO) Expert Committee on Food Additives (JECFA). Furthermore, Bet-CA-extracted pectin (68.08–77.47%) and ChCl-MaA-extracted pectin (65.85–73.06%) had higher GalA content than HCl-extracted pectin (61.54–68.89%). A significant increase in the GalA content was found in the pectins extracted from mango peels as the ultrasound power increased. Many researchers have reported that ultrasound/microwave irradiation may cause the degradation of the side-chain of pectin, thereby increasing the GalA content of the pectin extracted [19], [21]. It was found that (Ara + Gal)/Rha (10.60–13.25) of HCl-extracted pectin was significant higher than that of two DES-extracted pectins (7.21–10.99). In addition, the (Ara + Gal)/Rha value of the pectins extracted by different solvents was decreased with the increase of ultrasound power, and the lowest value dropped to 7.21. There results were in agreement with the reported findings of Zhang et al. (2013), who confirmed that ultrasound irradiation led to the degradation of the side chain of pectin [21]. As we known, galacturonic acid (GalA) usually acts as the main skeleton linear chain of pectin, and RG-I can be constructed by inserting a small amount of arabinose (Ara) in the GalA unit of pectin backbone. A remarkable decrease of RG-I was observed in all solvents-extracted pectins as the ultrasound power increased. Yu et al (2021) verified that ultrasound/microwave wave irradiation resulted in a considerable degradation of the pectin side chains under acidic environments [19]. Owing to the degradation of pectin side chains, the ratio of RG-I and (Ara + Gal)/Rha in the pectin extracted was significantly decreased. Homogalacturonan (HG), as the most abundant region of pectin, is consisted of long chains of linearly α-1,4-linked GalA residues. In this study, a remarkably increase of the HG region of all solvents-extracted pectins was seen with the increase of ultrasound power, which was consistent with the study of Hu et al. (2021) who reported that manosonication extraction evidently increased HG region of the pectin extracted from citrus peel wastes [47].

**Table 6:** The contents of monosaccharide and monosaccharide ratios of pectins extracted using different methods.

Pectin samples	Monosaccharide content (%, *w*/*w*)								Monosaccharide ratio			
	Man	Rha	GlcA	GalA	Glc	Gal	Xyl	Ara	Rha/GalA	(Ara + Gal)/Rha	HG	RG-I
HCl-U0W	0.60 ± 0.01Aa	2.15 ± 0.47Bc	1.32 ± 0.55Bd	61.54 ± 3.52Aa	11.26 ± 1.75Cd	14.97 ± 1.56Aa	1.11 ± 0.39Bc	2.54 ± 0.09Bd	0.036 ± 0.001Bc	8.16 ± 0.32Aa	59.39 ± 2.15Aa	18.23 ± 0.31Ad
HCl-U80W	0.61 ± 0.03Aa	1.59 ± 0.25Aa	0.51 ± 0.07Bc	62.14 ± 4.78Aa	14.30 ± 3.43Be	17.16 ± 3.47Ab	0.90 ± 0.10Bc	2.48 ± 0.17Bd	0.025 ± 0.001Ab	12.33 ± 1.03Bd	60.55 ± 1.78Ab	18.97 ± 0.76Bd
HCl-U240W	0.75 ± 0.01Ab	1.82 ± 0.18Ab	1.26 ± 1.19Bd	64.32 ± 4.39Aa	15.36 ± 1.88Cf	16.66 ± 2.78Aa	1.31 ± 0.49Bc	2.68 ± 0.45Cd	0.024 ± 0.002Ab	10.60 ± 0.71Cc	62.50 ± 2.67Ac	16.32 ± 0.57Cc
HCl-U400W	0.71 ± 0.09Bb	1.67 ± 0.36Aa	0.60 ± 0.23Bc	68.89 ± 4.21Ab	15.43 ± 3.02Cf	15.13 ± 2.68Aa	0.96 ± 0.44Ac	2.72 ± 0.78Cd	0.019 ± 0.005Aa	10.72 ± 0.98Cc	67.22 ± 3.13Ad	14.17 ± 0.91Bb
Bet-CA-U0W	0.80 ± 0.03Bb	1.79 ± 0.76Aa	0.32 ± 0.11Aa	68.08 ± 4.93Cb	9.87 ± 0.75Bc	17.30 ± 2.96Bb	1.54 ± 0.12Cc	2.32 ± 0.28Bd	0.024 ± 0.001Ab	10.99 ± 0.35Bc	66.29 ± 1.09Cd	18.06 ± 0.31Ad
Bet-CA-U80W	0.75 ± 0.05Bb	1.93 ± 0.51Bb	0.30 ± 0.05Aa	72.27 ± 5.49Cc	9.05 ± 2.04Ac	17.96 ± 2.01Ab	1.37 ± 0.25Cc	2.37 ± 0.72Bd	0.021 ± 0.000Ab	10.53 ± 0.28Ac	70.34 ± 1.17Cd	16.27 ± 0.07Ac
Bet-CA-U240W	0.78 ± 0.01Ab	2.14 ± 0.43Bc	0.18 ± 0.05Aa	74.73 ± 5.95Cc	10.28 ± 3.33Bd	17.44 ± 1.68Bb	1.08 ± 0.11Ac	2.04 ± 0.02Bd	0.021 ± 0.001Ab	9.09 ± 0.71Bb	73.59 ± 0.95Ce	14.05 ± 0.28Ab
Bet-CA-U400W	0.96 ± 0.02Bc	2.00 ± 0.21Bb	0.35 ± 0.04Ab	77.47 ± 7.74Cc	9.77 ± 1.86Ac	17.60 ± 2.47Bb	1.25 ± 0.38Bc	1.38 ± 0.21Ac	0.017 ± 0.002Aa	9.48 ± 0.63Bb	75.47 ± 0.74Cf	12.43 ± 0.15Aa
ChCl-MaA-U0W	0.58 ± 0.03Aa	2.29 ± 0.55Cc	0.30 ± 0.04Aa	65.85 ± 4.73Ba	8.61 ± 0.70Ab	17.65 ± 2.64Bb	0.27 ± 0.84Aa	0.72 ± 0.00Ab	0.036 ± 0.004Bc	8.02 ± 0.07Aa	63.56 ± 2.14Ac	19.25 ± 0.51Be
ChCl-MaA-U80W	0.56 ± 0.01Aa	1.94 ± 0.68Bb	0.30 ± 0.10Aa	66.52 ± 6.11Ba	9.23 ± 1.67Ac	19.56 ± 1.47Bc	0.53 ± 0.01Ab	1.04 ± 0.61Ac	0.028 ± 0.003Ab	10.63 ± 1.02Ac	64.58 ± 2.21Bc	19.77 ± 0.47Ce
ChCl-MaA-U240W	1.51 ± 0.03Bc	2.35 ± 0.63Cc	0.23 ± 0.08Aa	72.17 ± 4.34Bc	6.18 ± 0.32Aa	17.24 ± 2.10Bb	4.59 ± 0.11Cd	0.41 ± 0.06Aa	0.028 ± 0.004Ab	7.52 ± 0.37Aa	69.82 ± 1.82Bd	15.00 ± 0.95Bb
ChCl-MaA-U400W	0.60 ± 0.02Aa	2.15 ± 0.66Cc	1.32 ± 0.09Cd	73.06 ± 1.40Bc	11.26 ± 0.45Bd	14.97 ± 1.32Aa	1.11 ± 0.32Ac	2.54 ± 0.06Bd	0.029 ± 0.002Bb	8.14 ± 0.34Aa	70.91 ± 1.67Bd	17.51 ± 1.56Cd

### 3.4 FT-IR spectrum and thermal analysis

**Fig. 3:**
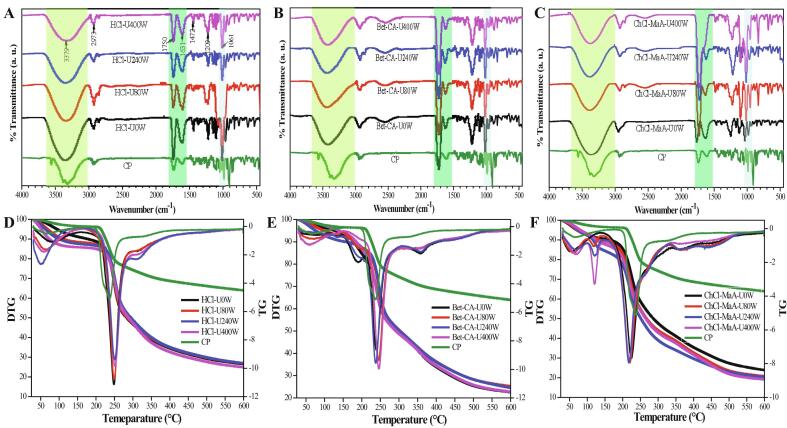
FT-IR spectrum (A–C) and thermal analysis (D–F) of commercial pectin (CP) and the pectins extracted from mango peel under different conditions.

FT-IR can be used as a quick and convenient method to investigate the functional groups of pectin polysaccharides. As shown in Fig. 3A-C, it can be found that the infrared spectra of the pectins extracted by different methods exhibited similar overall trends. Strong and wide peaks were found at 3379 cm^−1^, which were attributed to OH– stretching vibration group [2]. The weak band was observed at 2973 cm^−1^, which was credited to the C–H stretching vibration by the –CH, –CH_2_ and –CH_3_ groups of pectin polysaccharide polymer chains [22]. Two large peaks at 1750 cm^−1^ and 1631 cm^−1^ were typical absorbances in infrared spectra of pectin, which were due to the C=O stretching vibration of carbonyl functional groups (–COOCH_3_ or –COO^−^) [51]. In addition, the weak band at 1472 cm^−1^ indicated the presence of aliphatic or aromatic (C–H) plane deformation vibrations of methyl and methylene groups [52]. The peak at 1213 cm^−1^ was attributed to the C–O–C stretching vibration of pectin polymers chain structure [53]. The peak at 1061 cm^−1^ was associated to neutral arabinose-based glycans from RG-I. In general, the DE of pectin was correlated to the ratio of peak area at 1750 cm^−1^ to the sum of peak areas at 1750 cm^−1^ and 1631 cm^−1^. For two DES-extracted pectins, the peak area at 1750 cm^−1^ was stronger than that at 1631 cm^−1^, which led to a ratio greater than 70%. The results were consistent with the analysis results on pectins with high DE value (71.17%–87.06%). For HCl-extracted pectin, two peaks had similar intensities. In addition, it can be observed that the ratio of peak area at 1750 cm^−1^ to the sum of peak areas at 1750 cm^−1^ and 1631 cm^−1^ in three solvents-extracted pectins were evidently decreased as the ultrasound power increased (Fig. 3A-C). Hu et al. (2021) have reported that acoustic cavitation induced by ultrasonication can produce shear forces in the liquid around the bubble, which could break the C-O bond of the carboxyl groups, leading to a decreased degree of esterification value [47].

Thermal analysis is an important method to determine the physicochemical properties of pectin polysaccharides [54]. The results of TG and DTG are shown in Fig. 3D-F and Table S2. The weight loss process of mango peel pectins can be divided into three stages. The first stage was below 120 °C, in which TG curves of all extracted pectins and CP showed a slight decrease in weight, which was due to the loss of free and bound water. The weight loss rates of HCl-extracted pectin, Bet-CA-extracted pectin and ChCl-MaA-extracted pectin in the stage were 12.21%, 10.78%, 10.50%, respectively. The weight loss rate of mango peel pectins was significant higher than that of CP (4.50%). The second stage was between 220 °C and 350 °C, which was also known as the main weight loss stage, in which the decomposition (the rupture of carbon chain, hydrogen bonds and glycosidic bonds) of the pectin polysaccharide structures occurred. The weight loss rates of HCl-extracted pectin, Bet-CA-extracted pectin and ChCl-MaA-extracted pectin in the stage were 50.21%, 51.19% and 53.32%, respectively. The weight loss rate of all extracted pectins was even faster than that of CP (20.57%). Owing to biomass carbonization process, the weight loss rate of mango peel pectins in the last stage over 400 °C were 12.35%, 11.49%, 11.95%, respectively. For DTG curves, it was clear that maximum weight loss was obtained at 250.97 °C for HCl-extracted pectin, at 248.85 °C for Bet-CA-extracted pectin, at 226.19 °C for ChCl-MaA-extracted pectin and at 235.36 °C for CP, respectively. The results imply that HCl-extracted pectin and Bet-CA-extracted pectin have better thermal stability than ChCl-MaA-extracted pectin. In this study, it can be observed that ultrasound treatment significant varied the thermal stability of pectins. The thermal stability of pectins was decreased with the increasing of ultrasound power (Fig. 3D-F). Hu et al. (2021) have verified that the RG-I region had a positive correlation with the thermal stability of pectins [47]. Normally, high-branched (RG-I rich) pectin showed high thermal stability. From Table 6, the RG-I values of pectin extracted decreased with the increasing of ultrasound power. Therefore, the thermal stability of pectins was remarkably decreased as the ultrasound power increased.

### 3.5 Scanning electron microscopy

**Fig. 4:**
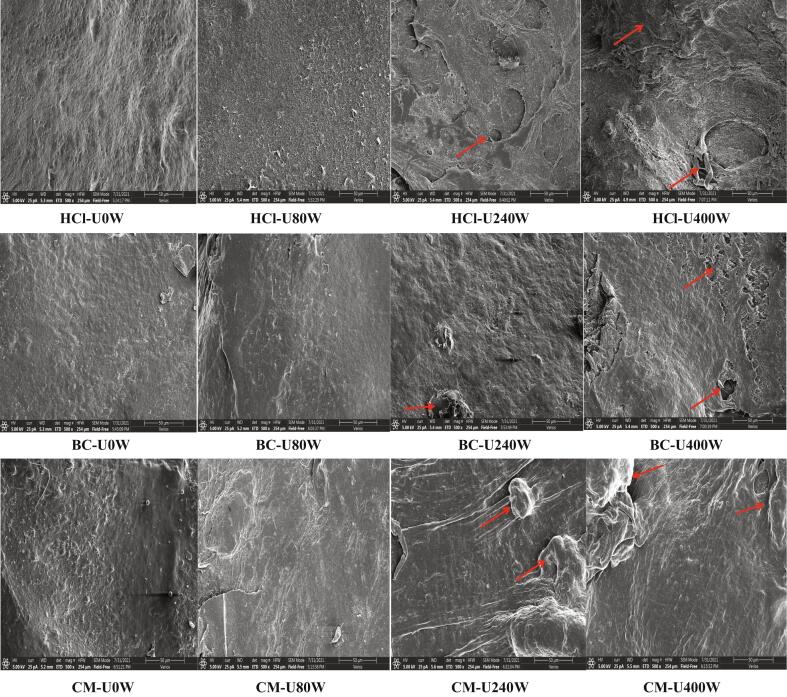
Scanning electron microscopy of the pectins extracted under different conditions.

The microstructures of pectin samples obtained by different methods were compared, as shown in Fig. 4. Without ultrasound treatment, pectin samples extracted by three different solvents had intact and smooth surface, in particular, the ChCl-MaA-extracted pectin had a relatively more rough surface than HCl-extracted pectin and Bet-CA-extracted pectin. After ultrasound treatment, significant microstructures discrepancy among the pectins could be observed. When ultrasound power was over 240 W, a cracking and wrinkled surface with heterogeneous flat micro-particles could be seen for all pectins samples extracted. In addition, it could be found that ChCl-MaA-extracted pectin had more smoother surface than HCl-extracted pectin and BC-extracted pectin, but HCl-extracted pectin had a cracking surface with more potholes than ChCl-MaA-extracted pectin and Bet-CA-extracted pectin. High intensity ultrasound waves remarkably affected the shape of pectins, resulting in cracking and potholes. Many researchers have confirmed that ultrasound cavitation may break the crosslinks between the pectin polysaccharides and other molecule matrix, leading to damage of the polymer network structure [50].

### 3.6 Rheological properties

#### 3.6.1 Apparent viscosity

**Fig. 5:**
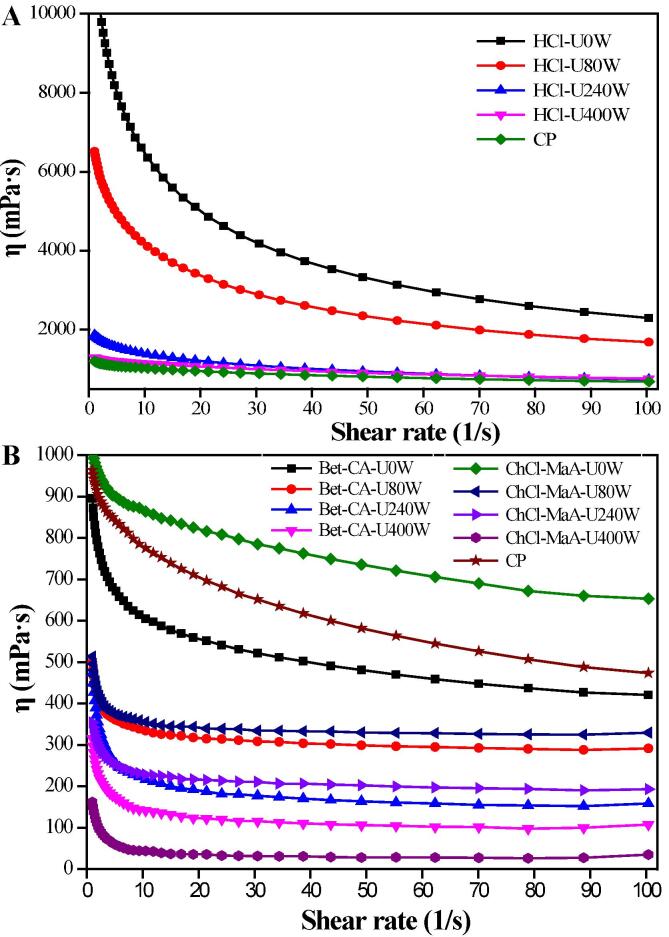
Apparent viscosity (η) of commercial pectin (CP) and mango peel pectins extracted using pH 2.5 HCl (A), Bet-CA and ChCl-MaA (B) under different ultrasound powers.

Extraction techniques including ultrasound or green solvents can alter the particular characteristics of pectin and contribute to its applications in food and cosmetic industries as gelling agents, stabilizers and emulsifiers [40], [48]. Fig. 5AB shows the flow behaviors of pectin solutions with concentration of 3%. The apparent viscosity of all pectin solutions decreased as the shear rate increased, which is indicative of a pseudoplastic fluid characteristic possibly owing to the gradual weakening of the pectin’s intermolecular strength. All HCl-extracted pectins showed higher apparent viscosity values than Bet-CA-extracted pectins and ChCl-MaA-extracted pectins (Fig. 5A). Yan et al. (2021) reported the apparent viscosity values of pectins were remarkably affected by pH value of the extraction agents [28]. Pectin extracted under acidic condition had higher viscosity value than that extracted under neutral or alkaline condition. Without ultrasound treatment, the apparent viscosity values of the pectins extracted ranked as follow: HCl-extracted pectin > ChCl-MaA-extracted pectin > Bet-CA-extracted pectin. Many studies have verified that DESs can damage the cross-linking bonds of polysaccharides macromolecule polymers, thereby decreasing their viscosity [40]. No matter which extraction solvent was used, the apparent viscosity of the pectins extracted gradual decreased with the increase of the ultrasound power. Some researchers have reported that the apparent viscosity of pectins is significantly dependent on their shear-thinning flow behaviors, and ultrasound waves can largely weaken the shear-thinning flow behaviors of pectins solution [28]. This finding might be due to the fact that compared with the pectins without ultrasound, the ultrasound-treated pectins with shorter side chains could cause less molecular entanglement, which led to the reduction in apparent viscosity. In particular, DES-extracted pectins retained stable and low viscosity values (Fig. 5B).

#### 3.6.2 Viscoelastic properties

**Fig. 6:**
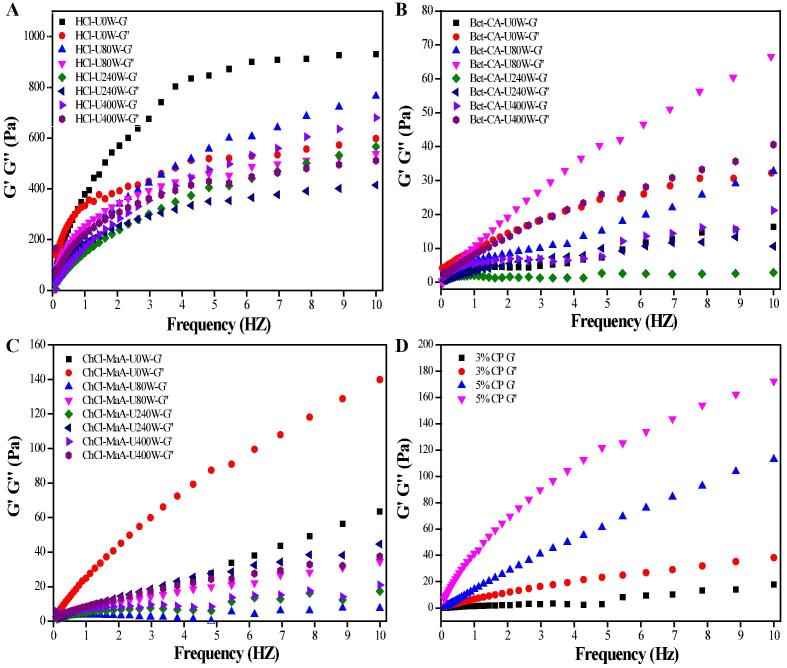
Changes in the storage modulus (G′) and loss modulus (G″) of mango peel pectins extracted using pH 2.5 HCl (A), Bet-CA (B) and ChCl-MaA (C) under different ultrasound powers and commercial pectin (D).

Generally, when the modulus ratio (G′/G″) of pectin is over 1, the pectin is in a colloid state. The changes in storage modulus (G′) and loss modulus (G″) of pectin solutions at the concentration of 3% are shown in Fig. 6. As expected, the values of G′ and G″ of all pectins extracted increased as the sweeps frequency increased from 0.1 Hz to 10 Hz. Sonication-synergistic DESs had significant effects on the viscoelastic properties of the pectins extracted. With respect to HCl-extracted pectin (Fig. 6A), the solution was in a colloid state (G′ > G″) during the specific frequency scope. However, the pectins extracted by DESs and commercial pectin (CP) were in a flow state (G′ < G″) (Fig. 6B-D). It was found that the values of storage modulus (G′) and loss modulus (G″) of HCl-extracted pectins were higher than those of DESs-extracted pectins and commercial pectin (Fig. 6A-D). Normally, the viscoelastic properties are related to the development of network structure and cross-linking combination among pectin chains [9]. Many researchers have verified that DESs can damage the cross-linking bonds of biomacromolecules, thereby decreasing their rheological properties. For DESs-extracted pectins, after ultrasound power treatment, it could be seen that G″ was always higher than G′ within the determined sweeps frequency range (0.1–10 Hz). The previous study confirmed that high intensity ultrasound irradiation led to the decline of the Mw and DE of the pectins extracted (Table 4), which resulted in rearrangement of pectin chains, increased molecular disentanglement, and a predominant viscous response (G′ < G″) [21].

## 4 Conclusions

This study comparatively analyzed the effects of ultrasound-synergistic eco-friendly natural DES and conventional HCl on the extraction yield, structural and physicochemical properties of the pectins extracted from mango peels for the first time. Two eco-friendly DESs, i.e. Bet-CA and ChCl-MaA with high pectin extraction efficiency, were screened, and the extraction conditions were further optimized by RSM. Under different ultrasound powers, two DESs showed higher pectin extraction yield than HCl. In addition, two DES-extracted pectins exhibited significant bigger molecular weight and larger particles size than HCl-extracted pectin. High intensity ultrasound treatment remarkably increased the extraction yield of low-ester pectins, but decreased their molecular weight and particles size. High content and DE value of GalA were observed for all pectins extracted from mango peels, but two DES-extracted pectins had significantly higher content and DE value of GalA than HCl-extracted pectin. HCl-extracted pectin had higher stability and viscosity than two DES-extracted pectin. Importantly, HCl-extracted pectin was in a colloid state, while two DESs-extracted pectin or CP were in a flow state. In short, sonication-synergistic natural DES significantly changed the structural and physicochemical properties of the pectins extracted from mango peels. The results provide a theoretical basis for seeking a high-efficient and eco-friendly extraction method of pectin from fruit wastes, and enhance our understanding of the relationship among extraction methods, structural and physicochemical properties of pectin.

## Declaration of Competing Interest

The authors declare that they have no known competing financial interests or personal relationships that could have appeared to influence the work reported in this paper.
