# Supplementary material for: The effect of sonication-synergistic natural deep eutectic solvents on extraction yield, structural and physicochemical properties of pectins extracted from mango peels
Source: Ultrason Sonochem. 2022 May 20;86:106045. doi: 10.1016/j.ultsonch.2022.106045 (PMC9136184; doi:10.1016/j.ultsonch.2022.106045)
Supplement: Supplementary data 1 [file mmc1.doc]

**The effect of sonication-synergistic natural deep eutectic solvents on extraction yield, structural and physicochemical properties of pectins extracted from mango peels**

Sijun Chen^1^, Leyan Xiao^1^, Songjie Li^1^, Tingyu Meng^1^, Lu Wang^1,2#^, Weimin Zhang^1,2#^

1. School of Food Science and Engineering, Hainan University, Haikou 570228, P. R. China;
2. Key Laboratory of Food Nutrition and Functional Food of Hainan Province, Hainan University, Haikou 570228, P. R. China;

Corresponding authors:

^#^**Lu Wang,** E-mail: [lwang@hainanu.edu.cn](mailto:lwang@hainanu.edu.cn)

**^#^Weimin Zhang,** E-mail: [zhwm1979@163.c](mailto:lwang@hainanu.edu.cn)om

**Supplementary Materials**

**Fig. S1.** The main factors of specified responses on the pectin yields from mango peels extracted by Bet-CA (A) or ChCl-MaA (B). Factor A, pH value of solvents; Factor B, liquid to solid ratio (L/S, mL/g); Factor C, water content in DESs (%); Factor D, temparature (℃); Factor E, extraction time (min).

**Fig. S2.** HPLC-DAD chromatogram (250 nm) for monosaccharide standards (A) and monosaccharide components of the pectins extracted from mango peels (B) under different conditions.

**Table S1** Experimental design and results of full factorial design experimental

| Run | Factors | |  |  |  | Respond |  | Factors | |  |  |  | Respond |
| --- | --- | --- | --- | --- | --- | --- | --- | --- | --- | --- | --- | --- | --- |
|  | pH | T (℃) | t (min) | L/S (mL/g) | WC (%) | Y_Bet-CA_ (%) |  | pH | T (℃) | t (min) | L/S (mL/g) | WC (%) | Y_ChCl-MaA_ |
| 1 | 1.70 | 70.00 | 120.00 | 30.00 | 90.00 | 19.59 |  | 0.40 | 90.00 | 120.00 | 30.00 | 30.00 | 17.64 |
| 2 | 2.70 | 70.00 | 80.00 | 30.00 | 90.00 | 10.43 |  | 1.40 | 90.00 | 120.00 | 30.00 | 90.00 | 13.92 |
| 3 | 2.70 | 70.00 | 80.00 | 15.00 | 30.00 | 7.54 |  | 0.40 | 90.00 | 80.00 | 30.00 | 90.00 | 18.02 |
| 4 | 2.70 | 90.00 | 80.00 | 30.00 | 30.00 | 9.73 |  | 1.40 | 70.00 | 120.00 | 30.00 | 30.00 | 8.36 |
| 5 | 1.70 | 90.00 | 80.00 | 15.00 | 30.00 | 11.80 |  | 0.40 | 70.00 | 80.00 | 15.00 | 90.00 | 9.90 |
| 6 | 1.70 | 90.00 | 120.00 | 30.00 | 30.00 | 2.38 |  | 1.40 | 90.00 | 80.00 | 30.00 | 30.00 | 11.71 |
| 7 | 2.70 | 70.00 | 120.00 | 15.00 | 90.00 | 13.14 |  | 1.40 | 90.00 | 120.00 | 15.00 | 30.00 | 11.55 |
| 8 | 1.70 | 70.00 | 80.00 | 15.00 | 90.00 | 16.44 |  | 0.40 | 70.00 | 80.00 | 30.00 | 30.00 | 9.17 |
| 9 | 1.70 | 90.00 | 80.00 | 30.00 | 90.00 | 18.73 |  | 0.40 | 70.00 | 120.00 | 30.00 | 90.00 | 7.79 |
| 10 | 1.70 | 90.00 | 120.00 | 15.00 | 90.00 | 17.89 |  | 0.40 | 90.00 | 80.00 | 15.00 | 30.00 | 13.71 |
| 11 | 2.70 | 90.00 | 120.00 | 15.00 | 30.00 | 14.42 |  | 0.40 | 90.00 | 120.00 | 15.00 | 90.00 | 21.26 |
| 12 | 2.70 | 90.00 | 120.00 | 30.00 | 90.00 | 14.59 |  | 1.40 | 70.00 | 80.00 | 15.00 | 30.00 | 4.09 |
| 13 | 2.70 | 70.00 | 120.00 | 30.00 | 30.00 | 10.08 |  | 1.40 | 70.00 | 80.00 | 30.00 | 90.00 | 9.31 |
| 14 | 2.70 | 90.00 | 80.00 | 15.00 | 90.00 | 16.12 |  | 1.40 | 90.00 | 80.00 | 15.00 | 90.00 | 7.42 |
| 15 | 1.70 | 70.00 | 120.00 | 15.00 | 30.00 | 13.24 |  | 1.40 | 70.00 | 120.00 | 15.00 | 90.00 | 9.23 |
| 16 | 1.70 | 70.00 | 80.00 | 30.00 | 30.00 | 8.24 |  | 0.40 | 70.00 | 120.00 | 15.00 | 30.00 | 9.42 |

Notes: T, extraction temperature (℃); t, extraction time (min); L/S, liquid to solid ratio (mL/g); WC, water content in DES (%)

**Table S2**

|  | Peak 1 (℃) | Weight loss (%) | Peak 2 (℃) | Weight loss (%) | Peak 3 (℃) | Weight loss (%) | Residual char yield at 600℃ (%) |
| --- | --- | --- | --- | --- | --- | --- | --- |
| HCl-U0W | 79.38 | 12.21 | 248.45 | 50.21 | 312.13 | 12.35 | 22.37 |
| HCl-U80W | 62.18 | 11.51 | 249.02 | 51.39 | 321.09 | 12.67 | 23.01 |
| HCl-U240W | 59.17 | 12.53 | 250.97 | 50.31 | 319.57 | 13.09 | 23.19 |
| HCl-U400W | 50.11 | 13.95 | 250.64 | 50.90 | 320.71 | 13.95 | 24.03 |
| Bet-CA-U0W | 65.57 | 10.78 | 238.87 | 51.19 | 356.34 | 11.49 | 21.07 |
| Bet-CA-U80W | 61.38 | 10.45 | 245.03 | 54.80 | 365.25 | 12.13 | 22.78 |
| Bet-CA-U240W | 59.87 | 9.76 | 248.85 | 55.58 | 361.97 | 11.07 | 22.65 |
| Bet-CA-U400W | 58.92 | 9.65 | 237.10 | 57.27 | 359.35 | 12.97 | 21.51 |
| ChCl-MaA-U0W | 53.4 | 8.87 | 226.19 | 53.32 | 357.10 | 11.95 | 25.37 |
| ChCl-MaA-U80W | 60.27 | 7.23 | 220.15 | 58.85 | 356.37 | 12.07 | 21.08 |
| ChCl-MaA-U240W | 68.66 | 7.51 | 218.47 | 53.63 | 361.41 | 12.98 | 21.13 |
| ChCl-MaA-U400W | 67.39 | 6.75 | 217.26 | 58.81 | 365.23 | 13.01 | 21.42 |
| CP | 66 | 4.51 | 235.36 | 20.57 | 354.28 | 11.95 | 64.31 |

Weight loss and residual char yield of the pectins extracted at distinct stages by thermal analysis
